# Editorial: Bioconversion and Biorefinery of C1 Compounds

**DOI:** 10.3389/fmicb.2021.778962

**Published:** 2021-10-28

**Authors:** Eun Yeol Lee, Fu-Li Li, Yu Wang, Pramod P. Wangikar, Michael Thomas Guarnieri, Guodong Luan

**Affiliations:** ^1^Department of Chemical Engineering (Integrated Engineering), College of Engineering, Kyung Hee University, Yongin-si, South Korea; ^2^Qingdao Institute of Bioenergy and Bioprocess Technology, Chinese Academy of Sciences (CAS), Qingdao, China; ^3^Key Laboratory of Systems Microbial Biotechnology, Tianjin Institute of Industrial Biotechnology, Chinese Academy of Sciences (CAS), Tianjin, China; ^4^Department of Chemical Engineering, Indian Institute of Technology Bombay, Mumbai, India; ^5^National Renewable Energy Laboratory (DOE), Golden, CO, United States

**Keywords:** C1 compounds, bioconversion, biorefinery, biocatalysts, green economy

The past decade has seen significant progress in the field of metabolic engineering and synthetic biology. The exponentially growing multi-omics data and technological advances in the development of efficient genetic manipulation tools and techniques have allowed scientists to explore and expand their understanding of microbial metabolisms and further develop sophisticated engineering strategies to realize the use of industrial “workhorses” and non-conventional microorganisms for sustainable bioconversion and biorefinery. There is of great interest for the research community in using C1 compounds (i.e., CO_2_, CO/syngas, methane, methanol) as the next generation feedstocks for microbial cell factories and biocatalysts to promote the sustainable development of a green economy (Figure 1). Considering lowering input costs is also a main consideration for successful business ventures, the use of inexpensive, abundant, and widely accessible C1 compounds is envisioned as a promising route for the sustainable production of fine chemicals, fuels, and other high-value products. Many C1 compounds are waste gases from industrial activities and may have detrimental effects on climate change upon emission into the atmosphere. Therefore, promoting the use of C1 compounds as renewable carbon feedstocks can greatly contribute to the reduction of anthropogenic emission of air pollutants.

**Figure 1:**
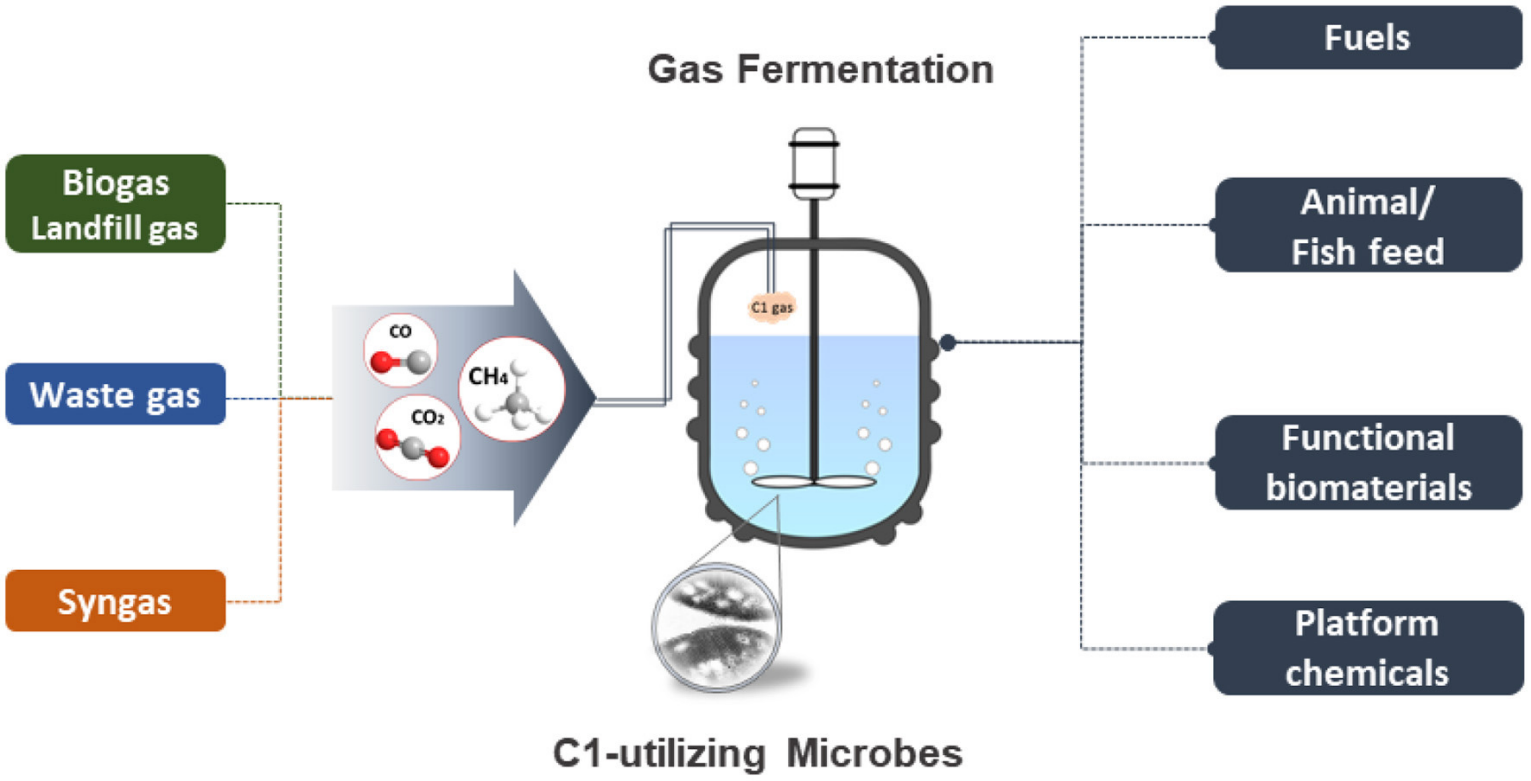
Bioconversion and biorefinery of C1 compounds.

In this Research Topic, a collection of articles including original research articles, reviews and minireviews specialized in C1 bioconversion and biorefining from leading research groups in the field is presented. Each article provides a state-of-the-art view of current metabolic engineering efforts, technical advances on contemporary genetic manipulation tools, and prospects in C1 bioconversion and biorefinery. The Research Topic focuses on the development of acetogens, cyanobacteria, methanotrophs, synthetic autotrophs, and synthetic methylotrophs as cell factories or biocatalysts for valorizing C1 compounds.

Compared to conventional chemical methods, bioconversion of C1 gases to liquids using microorganisms is an attractive approach to capture waste carbon for biorefining. In the current Research Topic, Sahoo et al. provide an overview of the core metabolic pathways of methanotrophs, their advantages, prospects, limitations, and consideration for further improvement. The capability of methanotrophs for bioconversion of methane into methanol at room temperature has been demonstrated before. However, the constant expression of methanol dehydrogenase reduces the yield of methanol bioconversion. To address the issue, Ito et al. developed a methanol dehydrogenase (MxaF) knockout mutant of *Methylosinus trichosporium* OB3b to enable switching between methanol accumulation mode and cell growth. Despite the low conversion efficiency, the capability of mutant for methanol accumulation can be maintained longer than wild-type and does not require inhibitors to accumulate methanol. Another recent advance for metabolic engineering of methanotrophs is the development of efficient electroporation systems for chromosomal deletion and integration for heterologous expression. Although genetic manipulation tools for methanotrophs have been explored for a while, Hu et al. have developed and optimized electroporation parameters for gene deletion and heterologous gene expression through electroporation of linear DNA fragments, increasing the maximum electroporation efficiency by 10-fold.

Valorization of CO_2_ into high-value products is an important aspect of C1 bioconversion, thus significant efforts have been exerted to improve and expand host organisms and genetic manipulation tools. Cyanobacteria and microalgae are the two groups of hosts commonly employed to produce high-value products from CO_2_. Despite being the largest phylum of prokaryotes, only a handful of model cyanobacteria have been studied and employed for biotechnological applications. To further expand the range of available model cyanobacteria, Chenebault et al. have developed a genetic toolbox for the robust unicellular cyanobacterium *Cyanothece* PCC 7425 and further demonstrated the production of limonene in this species. In another study, Gupta and Srivastava highlighted the combinatorial effects of promoters and ribosome binding sites on growth performance and glycogen production of *Synechococcus* sp. PCC 7002 overexpressing a sodium-dependent bicarbonate transporter, SbtA. Host development is also another approach for improving the capabilities of cell factories. For scaling up the production from cyanobacteria for outdoor cultivations, other traits such as high light, high temperature and salt tolerances are desirable. Zhang et al. proposed and demonstrated a convenient and markerless strategy for rapidly improving high light and high temperature tolerances of an important cyanobacterial chassis *S. elongatus* PCC 7942 through an introduction of a point mutation (C252F) in ATP synthase α subunit. Cui et al. have successfully improved salt tolerance of a promising cyanobacterium *S. elongatus* UTEX 2973 by redirecting and enhancing carbon flux toward a glucosylglycerol biosynthetic pathway. Computational analysis is a valuable tool for culture conditions optimization. Vasile et al. constructed a model framework that encompasses multi factors to relay key physiological parameters in the cultivation environment. These studies expand the current strategies to engineer more efficient cyanobacterial chassis cells. In the case of microalgae, owing to their unique properties and high potential applications in nutraceutical and pharmaceutical industries, they have gained considerable interest for commercialization. Yet, the lack of understanding of regulatory networks remains a major obstacle. To tackle this problem, Mariam et al. employed omics analysis and media engineering to gain new insights on the methyl erythritol phosphate (MEP) pathway and crosstalk between different metabolic pathways in *Botryococcus braunii*. The study proposes a new strategy to enhanced production without compromising growth.

Several articles in the current Research Topic focused on improving solventogenesis of acetogens, a group of bacteria capable of utilizing CO, CO_2_ and H_2_ *via* Wood–Ljungdahl pathway. In a recent study, Han et al. have reported the dramatic effects of a combination of trace metals on the production of higher alcohols in syngas fermentation. The yields obtained from the modified composition were 10-fold higher than the original composition, with the maximum concentrations of accumulated ethanol and butanol reaching 2.0 and 1.0 g/L, respectively. In another study, He et al. demonstrated the effects of process parameters on the production of bioethanol from H_2_/CO_2_ by solventogenesis using anaerobic granular sludge. These studies provide important insights on solventogenesis optimization by adjusting operational temperature and trace metal composition.

The recent emergence of synthetic methylotrophs and autotrophs is also of equal importance to facilitate a sustainable bioeconomy. The development of synthetic biology and efficient genetic tools for industrial workhorses have made it possible to introduce complex carbon-fixing modules to heterologous hosts. Furthermore, with a better understanding of the natural carbon fixation pathways of microorganisms, developing efficient designs for synthetic C1 fixation pathways is no longer improbable. In a review article from Liang et al., the recent advances, prospects, and main challenges in developing efficient synthetic autotrophic microorganisms are thoroughly discussed. Novel one-carbon assimilation pathways were investigated in the work of Mao et al.. In this work, a comb-flux balance analysis algorithm was employed to predict novel and carbon-conserving formaldehyde assimilation pathways that are independent of additional energy and/or reducing power. The work demonstrated a systematic approach to design C1 assimilation pathways using artificial aldolases, in which one novel pathway named the glycolaldehyde-allose 6-phosphate assimilation pathway achieved a high carbon yield of 94% *in vitro*. A major challenge for further development of synthetic methylotrophs is the cytotoxicity from substrates and intermediates such as methanol and formaldehyde. While there are reports on successful constructions of synthetic methylotrophs, poor growth rates and biomass accumulation are common observations. Bennett et al. attempted to address this issue by conducting chemical mutagenesis and adaptive laboratory adaptation on an *Escherichia coli* methylotroph. The authors found a connection between common mutations in the 30S ribosomal subunit proteins and methanol tolerance. The findings provide insight into a novel methanol tolerance mechanism for synthetic methylotrophs and further advance the development of this field. However, the improved methanol tolerance is specifically attributed to methanol, not formaldehyde. It remains to be seen how to improve formaldehyde tolerance of synthetic methylotrophs.

Overall, there is no doubt that bioconversion and biorefinery of C1 compounds using metabolically engineered microorganisms present a promising route to promote sustainable development of the bioeconomy in the future. With the development of more efficient C1 biocatalysts, a new range of biotechnical applications and perspectives can be opened. Although there is still a long road ahead, it is our belief that the series of articles in the Research Topic provide a contemporary overview and multiple metabolic engineering strategies that can help to pave the way for industrial applications of C1 compounds in the future.

## Author Contributions

EL drafted this editorial. F-LL, YW, PW, MG, and GL edited the final text. All authors approved the final version.

## Funding

The C1 Gas Refinery Program through the National Research Foundation of Korea (NRF) funded by the Ministry of Science and ICT (2015M3D3A1A01064882), the National Key R&D Program of China (2018YFA0901500 and 2018YFA0903600), and the Youth Innovation Promotion Association of Chinese Academy of Sciences (2021177) are appreciated. MG was supported by the US Department of Energy, Office of Energy Efficiency and Renewable Energy under Agreement No. 26680.

## Author Disclaimer

The views and opinions of the authors expressed herein do not necessarily state or reflect those of the United States Government or any agency thereof. Neither the United States Government nor any agency thereof, nor any of their employees, makes any warranty, expressed or implied, or assumes any legal liability or responsibility for the accuracy, completeness, or usefulness of any information, apparatus, product, or process disclosed, or represents that its use would not infringe privately owned rights.

## Conflict of Interest

The authors declare that the research was conducted in the absence of any commercial or financial relationships that could be construed as a potential conflict of interest.

## Publisher's Note

All claims expressed in this article are solely those of the authors and do not necessarily represent those of their affiliated organizations, or those of the publisher, the editors and the reviewers. Any product that may be evaluated in this article, or claim that may be made by its manufacturer, is not guaranteed or endorsed by the publisher.

